# Using a Stakeholder Analysis to Implement the Belgian One Health National Report for Antimicrobial Use and Resistance

**DOI:** 10.3390/antibiotics13010084

**Published:** 2024-01-16

**Authors:** Mickaël Cargnel, Moira Kelly, Hein Imberechts, Boudewijn Catry, Maria-Eleni Filippitzi

**Affiliations:** 1 Coordination of Veterinary Activities Service, Infectious Diseases in Animals Department, Sciensano, 1050 Brussels, Belgium; 2 Healthcare-Associated Infections and Antimicrobial Resistance Service, Epidemiology and Public Health Department, Sciensano, 1050 Brussels, Belgium; moira.kelly@sciensano.be (M.K.); boudewijn.catry@sciensano.be (B.C.); 3 Strategy and External Positioning, Sciensano, 1050 Brussels, Belgium; hein.imberechts@sciensano.be; 4 Faculty of Medicine, Université Libre de Bruxelles, 1070 Brussels, Belgium; 5 Animal Production, Ichthyology, Ecology and Protection of the Environment Department, Health Sciences School, Faculty of Veterinary Medicine, Aristotle University of Thessaloniki, University Campus, 54124 Thessaloniki, Greece; mefilippi@vet.auth.gr

**Keywords:** stakeholder analysis, BELMAP, antimicrobial resistance, antimicrobial surveillance

## Abstract

(1) Background. Antimicrobial resistance (AMR) poses a substantial global health threat with profound economic implications. Acknowledging the imperative for a One Health (OH) strategy to combat this menace, Belgium introduced an annual national OH report, known as the “BELMAP report,” encompassing antimicrobial use (AMU) and AMR, with the first edition completed in 2021. The integration of innovations for the healthcare system demands a meticulously planned process. (2) Methods. We introduced a three-step stakeholder analysis (SA) as a prospective framework for navigating this new report process, fostering complementary collaboration, pinpointing obstacles, suggesting approaches to overcome them, and facilitating national policy development. The SA unfolds in three steps: stakeholders identify and list their relevant activities, assess their positions regarding the BELMAP report, and complete “actor mapping” of national AMR and AMU stakeholders. (3) Results. Stakeholder identification reveals a fragmented landscape of AMR and AMU activities across Belgium. Assessment of stakeholder positions uncovers diverse expectations, collaborative challenges, and resource considerations. “Actor mapping” identifies key stakeholders, emphasizing the importance of high-interest and high-power actors. (4) Conclusions. This SA approach not only provides insights into the present stakeholder landscape in Belgium, it can also serve as a blueprint for other countries in the process of developing OH reports.

## 1. Introduction

The development and spread of antimicrobial resistance (AMR) poses a serious global health risk, and its consequences have far-reaching economic impacts due to treatment failures as well as increased rates of morbidity and mortality in both human and animal populations. It is estimated that at least 700,000 people lose their lives annually due to treatment failures resulting from AMR, and this number is projected to skyrocket to 10 million people per year by 2050. This trend would come at an exorbitant cost of USD 100 trillion to the global economy [1]. It is evident that urgent and coordinated efforts at national and international levels are essential to address this critical issue and safeguard the efficacy of antimicrobial agents for current and future generations.

The World Health Organization (WHO) strongly advocates for the enhancement of national multi-disciplinary systems and cross-sector approach, embracing the “One World, One Health” approach, as a crucial measure in effectively addressing complex health challenges [2]. Regarding AMR, the One Health (OH) approach acknowledges that antimicrobial resistance is a health issue that extends beyond single sectors and demands expertise and concerted efforts from multiple interconnected disciplines [3]. By fostering collaboration among experts in human medicine, veterinary medicine, and the environment, the OH approach in tackling AMR can create a synergistic, integrated, and mutually beneficial system. This system can better understand and manage these health threats which transcends the human–animal–environment interface [3,4,5].

In 2015, the WHO developed the Global Action Plan on AMR [6]. This comprehensive plan urged all countries to establish a National Action Plan (NAP), outlining a framework with five strategic objectives to combat AMR effectively. One of these strategic objectives is the establishment of cross-sector surveillance. This process involves gathering AMR and AMU data, enabling the monitoring of trends, detecting emerging resistance, and providing essential data for risk analysis and policy recommendations [7]. In response to the WHO Global Action Plan, Belgium took a significant step by adopting a One Health National Action Plan (NAP) for AMR in 2020 [8]. The NAP aimed to improve coordination, collaboration, and communication among different data providers and sectors. As part of this effort, one goal set in the NAP was to develop an annual national OH report that focuses on both AMR and AMU, known as the BELMAP report. The BELMAP report brings together available data from the human, animal, food, and environmental sectors, thus fostering a unified approach and providing comprehensive insights into the status of AMR and AMU in the country, similar to approaches from leading countries in the field, such as Denmark, France, and the Netherlands.

With the primary goal of supporting the practical implementation of the OH framework in Europe for health threats including AMR, the One Health European Joint Programme (OHEJP) (2018–2023) was launched. This program facilitated the establishment of new OH activities and resources through “integrative action” and “joint research” projects [9]. One of these projects was the One Health Surveillance Initiative on Harmonization of Data Collection and Interpretation (ORION) project; this study was conducted within that framework.

The overarching objective of this study was to foster a collaborative OH approach between all the sectors in Belgium that are involved in activities related to AMR/AMU surveillance while developing the “BELMAP report”. To achieve this, a stakeholder analysis (SA) was performed. Stakeholder analysis is a process of identifying and understanding individuals or groups affected by a project/policy decision to inform decision-makers about involved stakeholders’ interrelations, behaviours, interests, and their potential to intervene in the project decision [10]. In this way, SA can support the implementation of a policy decision and can also provide valuable recommendations to policymakers on how to effectively consider all stakeholder perspectives [10,11,12,13,14,15]. To our knowledge, this is the first time that the SA method has been employed to address the implementation of a multi-disciplinary project within the health sector, and specifically, in the One Health domain.

## 2. Results

### 2.1. Questionnaires and Participants

The first questionnaire (Questionnaire S1) garnered 45 responses, yielding a response rate of 21%, and the second questionnaire (Questionnaire S2) was completed by 33 participants, resulting in a completion rate of 15%.

### 2.2. Stakeholder Analysis

#### 2.2.1. Step 1: Stakeholders Identification and activities

The 45 responses collected in Questionnaire S1 were provided by stakeholders belonging to 28 different organizations (Table 1). The detailed results of the organizations identified together with info on their AMR and AMU activities are available online at https://mika147852.wixsite.com/onehealthbelgium (accessed on 5 January 2024) and are summarized in Table 2 (AMR) and Table 3 (AMU).

Concerning AMR, there are three key organizations involved in the publication of original reports related to AMR online. These organizations are Sciensano and the two regional veterinary laboratories, namely, the Regional Association for Animal Health and Identification (ARSIA) and the Dierengezondheidszorg Vlaanderen (DGZ). The reports cover a range of targets, including feed, various types of food (e.g., fresh meat), food-producing animals (such as cattle, poultry, pigs, horses, and small ruminants), human strains and clinical samples.

ARSIA and DGZ primarily focus on analysing samples obtained from diseased animals. In contrast, Sciensano collects different samples to adhere to national and European monitoring programs, such as European Decision 2020/1729, which pertains to the monitoring and reporting of antimicrobial resistance in zoonotic and commensal bacteria. Sciensano’s AMR activities extend to encompass the domains of food and feed. Nevertheless, it is important to note that the specific bacteria targeted within these various sectors may differ. Notable bacteria with characterized resistance profiles in both human and animal contexts include *Enterococcus faecalis*, *Enterococcus faecium*, *Pseudomonas*, *Salmonella* spp., *Staphylococcus aureus*, and *Streptococcus*. However, variations exist with respect to the antimicrobials under investigation.

In Belgium, the reporting of AMU involves the active participation of four key stakeholders. Sciensano is responsible for collecting data from both acute care hospitals and long-term care facilities. This institute contributes valuable insights into the use of antimicrobials in these healthcare settings. Ghent University (UGent) focuses on gathering data related to AMU in livestock and pets, whereas the Knowledge Centre on Antibiotic Use and Resistance in Animals (AMCRA) concentrates on the livestock industry. Furthermore, the University of Antwerpen takes part in the Global Point Prevalence Survey of Antimicrobial Consumption and Resistance in hospitals and healthcare centres. Currently, only the reports related to AMU in animals are publicly available in a yearly report by AMCRA, providing updated insights into antimicrobial usage trends in the animal sector in Belgium.

#### 2.2.2. Step 2: Assessment of Stakeholder Positions Regarding the BELMAP Report

- Method 1: Stakeholder expectations identification

The expectations from the BELMAP report, as articulated by the 41 professionals who responded to this question in Questionnaire S1, have been systematically compiled (raw answers in Results S1). These responses have been categorized into themes of ideas, providing a snapshot of the prevalent expectations. The five most prevalent themes that have been identified are presented in Table 4, together with a description of the theme summarizing the answers received.

The first theme, Collaboration and partnerships, emphasizes collaboration across sectors to address AMR and AMU in both human and veterinary medicine. It advocates for a comprehensive approach involving governments, institutions, professionals, and the public. The second theme, Data collection and analysis, indicates that gathering comprehensive data on AMR and AMU from various sources, including human and animal health sectors and different geographical levels, is crucial for informed decision-making. The theme Clear guidelines and recommendations underscores the need for clear guidelines in human and veterinary medicine to promote responsible AMU. The objective is to provide practical, evidence-based guidance to reduce unnecessary antibiotic usage and mitigate AMR. The theme Awareness and education aims to raise awareness and to educate both professionals and the public about AMR to encourage responsible antibiotic use and collective efforts against resistance. The last theme, Practical implementation, is about turning guidelines into action by implementing practical steps, including individualized treatments, exploring alternatives to antibiotics, and incorporating diagnostic tools.

- Method 2: Analysing stakeholder collaboration employing strengths, weaknesses, opportunities, threats (SWOT) analysis

The strengths, weaknesses, opportunities, threats (SWOT) analysis was performed based on 42 respondents (33 full answers, nine partial answers, two no answer and one answer that was removed as it was out of the scope (gave nonsensical answers)). The results of the SWOT analysis are shown in Table 5, which presents the five most prevalent themes expressed by the respondents.

The main strength (S) of the BELMAP report, as expressed by the respondents, is the collaboration and complementarity that can result from a OH collaboration between numerous stakeholders working in different sectors. The main weakness (W), according to the respondents, is the fragmentation of competencies. The stakeholders expressed a possible difficulty in producing a report due to the involvement of numerous stakeholders with different interests and competencies, and due to a lack of methodological harmonization. The BELMAP report is seen as an opportunity (O) for the respondents to increase awareness of AMR. Finally, the main threat (T) to the production of this report are resource limitations and underfunding.

- Method 3: Focus group interview

A total of eight scientists participated in the focus group interview. For the *verbatim* transcription, please refer to Supplementary materials (Results S2). The different themes highlighted during the conversation are presented and described in Table 6. These themes are: involvement and participation, mandate and authority, collaboration and data comparison, defining content and scope, short and policy-guiding report.

The participants engaged in the discussion were: Veterinary Epidemiology 1, Veterinary Epidemiology 2, Services of the Managing Direction, Food Pathogens, Bacterial Diseases, Veterinary Bacteriology, Mycology and Aerobiology, and One Health Coordinator.

The dialogue concluded with participants concurring on the importance of securing a clear mandate from authorities, engaging external stakeholders, and outlining report content. The value of collaboration, discussions with external stakeholders, and harmonizing methodologies was reiterated as pivotal in creating a meaningful, actionable report.

#### 2.2.3. Step 3: Actor Mapping

**Figure 1:**
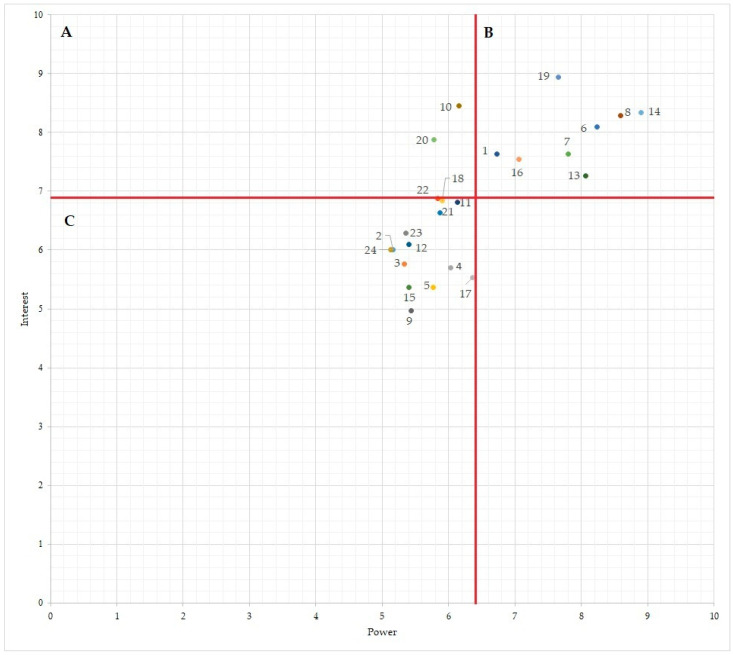
Actor-map grading the stakeholders based on their power and interested in publishing a common national one health antimicrobial/antimicrobial usage or consumption report. Legend. Numbers in the figure correspond to specific stakeholders (refer to Table 7 for details). Region A integrates stakeholders with high interest and low power. Region B: stakeholders with high interest and high power. Region C: stakeholders with low interest and low power. The global mean power score was represented on the y-axis and the global mean interest score on the x-axis by red lines.

Thirty individuals provided complete responses to Questionnaire S2. The mean power and interest scores are found to be 6.42 and 6.85 respectively, with a maximum possible score of 10 (Figure 1).

When evaluating the power scores reported, several stakeholders emerge as predominant actors, showing significant influence in the implementation of the BELMAP report. The Minister of Health, the Food Agency for the Safety of the Food Chain (FASFC), and the Federal Agency for Medicines and Health Products are deemed to wield authority and decision-making capacity (top three highest power scores). Conversely, the Belgian Feed Association, Certification/label/sector/professional associations (lowest power score), and others with lower power scores are situated at a different echelon of influence.

Shifting focus to interest scores, Sciensano, the Knowledge Centre of Antibiotic Use and Resistance in Animals (AMCRA), and the Minister of Health take the lead, demonstrating intense engagement and investment in the topics surrounding AMR and AMU. Meanwhile, stakeholders such as food retailers, the National Belgian Federation of Slaughterhouses (FEBEV), and the farmers’ association could manifest relatively the lowest levels of interest.

Based on the combined scores of power and interest, a subset of stakeholders emerge as “key players”, with high power and high interest (Figure 1: region B). These actors exhibit dual high power and interest. Noteworthy examples among these key players include the Belgian Antibiotic Policy Coordination Committee (BAPCOC), the Federal agency for medicines and health products, the Food Agency for the Safety of the Food Chain, the Federal Public Service, the Minister of Agriculture, the Minister of Health, the National Institute for Health and Disability Insurance, and Sciensano. Their confluence of power and interest position them as pivotal contributors to the BELMAP report but also to shaping antimicrobial resistance and usage policies.

Conversely, three stakeholders stand out with high levels of interest but comparatively lower power in terms of influence in policy formulation and decision-making (Figure 1: region A). These include AMCRA, veterinary practitioners, and universities.

The other stakeholders, including the Belgian Feed Association, Certification/label/sector/professional associations, Farmers association, Food retailers, and Milk sector organisations/labs, display low levels of both power and interest (Figure 1: region C). These stakeholders appear to have limited influence and engagement in the context of the Belmap report discussions. No stakeholders are identified with high power and low interest.

## 3. Discussion

This study employed a stakeholder analysis (SA) to facilitate the drafting of the first version of the Belgian national One Health report for AMR and AMU, known as the BELMAP report. Although the SA has received growing recognition in recent years as an integral component of health innovation planning processes [16], this is the first time this methodology has been employed to facilitate a national OH action. This analysis can serve as a potential example for other countries in the process of developing a national OH report.

The SA’s first step, the ‘Stakeholders identification and activities’, was useful to identify AMR and AMU activities in Belgium. It revealed a landscape characterized by a certain level of fragmentation and data dispersion in addressing AMR and, to a lesser extent, AMU at the national level. Fragmentation in the health sector can be defined as “*as lack of coordination between the different levels and settings of care, duplication of services and infrastructure, unutilized productive capacity, and health care provided at the least appropriate location*” [17]. Our investigations identified various independent initiatives (e.g., AMR reports) undertaken by diverse players without a clear national coordination, often working in isolation without a common goal. A recent review of European AMR surveillance systems [18] resulted in similar findings, with numerous local and national systems that lacked coordination, harmonization in information-sharing with (inter)national networks. This fragmentation specifically poses a challenge in managing complex health concerns like AMR. It impedes the capacity of identifying potential gaps or redundancies in the ongoing national AMR and AMU monitoring activities. For example, the results of the SA have highlighted a potential deficiency in data related to AMR in different matrices of the environment sector. Indeed, a holistic surveillance approach should include the essential role of the environment as a reservoir for AMR and the transmission of resistance genes or germs to both humans and animals [19]. To contribute to reducing this fragmentation, an official website could be created, aiming to serve as a centralized hub where stakeholders can share and access comprehensive data about their AMR and AMU initiatives, facilitating a more holistic understanding of the landscape. This could be a first step in data harmonization and alignment of data. Additionally, the website could aim to provide policy makers and the public with clear and accessible information on Belgian stakeholders’ AMR and AMU activities, with the ultimate goal of fostering cross-sectoral collaboration. It is imperative to plan a broad communication campaign to raise awareness among stakeholders of the data that will be available but scattered over various sources and to persuade them of the added value of the website in centralizing AMR and AMU data.

Analysing stakeholder positions (SA step 2) in the development of a national report on AMR and AMU has been vital because it enabled the understanding of the stakeholders’ requirements, the collaborative process, and helped to identify potential challenges, conflicts, barriers. This process is essential to elaborate the future BELMAP report that should meet all stakeholders’ expectations and needs. To complete this step, this study used three complementary methods, namely identification of stakeholders’ expectations, SWOT analysis, and focus group interviews. Determining stakeholders’ expectations (step 2, method 1) was essential in ensuring a balanced representation of all stakeholder perspectives concerning the BELMAP report. The insights gained from this step (e.g., the BELMAP report needs to provide practical recommendations for the field actors) must be considered during the drafting of the report to avoid future cognitive dissonance between stakeholders. This information should also be considered to construct an effective communication campaign that can highlight the necessity, legitimacy, and rationale behind the BELMAP report, stimulating stakeholder interest. When the report fails to align with the concerns, expectations, values, and needs of stakeholders, it may lead to cognitive dissonance [20]. Therefore, it is crucial for all pertinent stakeholder groups to either explicitly communicate their expectations or be given the chance to express their opinions throughout the entire project duration.

We considered that the assessment of the level of collaboration between stakeholders using a SWOT analysis (step 2, method 2) was crucial before the beginning of the project. It helped to identify the strengths and weaknesses in the development of the report, opportunities for improvement, potential threats, to guide decision-making, and ultimately to contribute to the report’s success by enhancing the collaborative process. The main weaknesses and threats we identified were linked to the possible difficulty for the different sectors (e.g., human medicine, veterinary medicine, food science) to join their effort and share and analyse data together. This may reduce the involvement of numerous stakeholders, and the project success.

The focus group interview (step 2, method 3) was valuable in identifying challenges and concerns among AMU/AMR scientists/practitioners involved in drafting the preliminary BELMAP report drafting. In our research, the strength of employing focus group interviews is evident, fostering a nuanced understanding of our topic. Through dynamic group discussions, we identified viewpoints and emerging trends, surpassing the limitations of traditional questionnaires. Furthermore, focus group interviews permit real-time interaction and the clarification of responses, which allowed us to delve deeper into participants’ thoughts.

When writing a national AMR/AMU report, it is essential to identify contributors as well as their roles and contributions, and to determine the parties responsible for leading communication between different entities and possessing the necessary resources, power, and leadership to resolve any issues encountered [21]. This is why the third step of the SA, actor mapping, is a valuable tool for this purpose as it helps to find stakeholders who may have the required resources to resolve the concerns identified in SA step 2. Stakeholders who are identified with both high interest and high power are pivotal players in the project’s success. They often control critical resources, possess substantial decision-making authority, and are effective in risk management. Their deep involvement and influence make them advocates for the project and vigilant monitors of its progress, contributing to timely adjustments and alignment with evolving requirements. These authorities should step in to set priorities aligned with everyone’s needs and interests and make necessary decisions. Stakeholders with low power and interest necessitate monitoring without overwhelming them with excessive information. Conversely, stakeholders with high interest but limited power should be kept informed to ensure their needs are met for the initiative’s success [10,22]. Notably, our study revealed unexpected classifications in the actor map. For example, the two regional veterinary laboratories were categorized as having low interest in the BELMAP report, despite being us considering them to have high interest. These laboratories play an active role in monitoring AMR in pathogenic bacteria in livestock, making their active collaboration essential and their contributions indispensable, given their extensive experience in laboratory testing and monitoring. Investigating the reasons behind their low interest score is crucial as the alignment between expected and actual interest and power is fundamental to preventing inadequate involvement and support, which could undermine the initiative. Sciensano, the national reference laboratory, was categorized with high power (meaning high capacity to influence the decision-making). This is surprising because this lab will contribute to the BELMAP report with the same level of influence as other data providers.

Lastly, it is crucial to emphasize that the insights gleaned from this SA represent the present situation. It is imperative to stress that SA should not be a singular undertaking but an ongoing and iterative process. The continuous monitoring of stakeholder dynamics and the adaptive refinement of project management strategies are essential in this endeavour.

## 4. Materials and Methods

**Figure 2:**
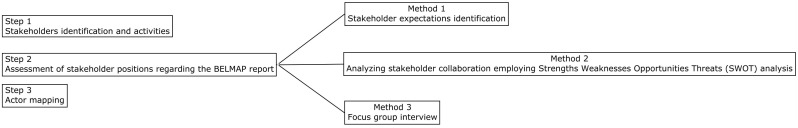
Stakeholder analysis workflow.

To address the objective of this study, a SA was conducted (see Section 4.2) and will be described step by step. Figure 2 shows the detailed outline of the methodology used in this study.

### 4.1. Questionnaires and Participants

Two distinct questionnaires, Questionnaire S1 and Questionnaire S2, were created to capture the information essential for conducting a SA, as elaborated upon in Section 4.2. We have opted to split the questions into two separate questionnaires, used at two different times. This approach allowed us to divide the total response time in half and reduced the likelihood of respondents discontinuing due to the length of the survey. These questionnaires were thoughtfully administered to professionals and experts working in the area of public health and AMR. The initial cohort consisted of 210 professionals and experts who willingly shared their email addresses with the Federal Public Health Service (FPHS) during the “Belgian One Health Network, Launching Event” held on 5 November 2019. This event served as a pioneering initiative, aimed at advancing the principles of One Health and fostering collaboration among individuals engaged in this domain across Belgium. Subsequently, the list of participants was reviewed by two experts from the FPHS and three experts specializing in antimicrobial resistance from Sciensano, which is the national reference laboratory for AMR in public and animal health in Belgium. This comprehensive evaluation ensured the inclusion of all major stakeholders. Notably, this resulted in the incorporation of eight additional stakeholders onto the list. To guarantee confidentiality, the contact (email) list was not disclosed to the authors of this study. Instead, the identified professionals (218 in total) were directly furnished with Questionnaire S1 and Questionnaire S2 (Questionnaire S2 was sent two months after Questionnaire S1), courtesy of the FPHS. This approach ensured a confidential process for data collection.

### 4.2. Stakeholder Analysis (SA)

The stakeholder analysis approach involves a systematic process of three essential steps, as outlined by Gilson [23] and Roberts [24]. These steps are:

Step 1: Stakeholder identification and activities. The first step entails identifying the pertinent groups and individuals (the stakeholders) who hold relevance to the policy matter under consideration.

Step 2: Assessment of stakeholder positions. In the second step, an evaluation is conducted to determine the opinion of each stakeholder regarding the issue, named in this study as the “Assessment of stakeholder positions regarding the BELMAP Report”.

Step 3. Actor mapping. The third step assesses the relative influence and power wielded by each stakeholder concerning the issue.

#### 4.2.1. Step 1: Stakeholders Identification and Activities

In the initial phase of our study (Step 1), we identified and catalogued all stakeholders engaged in activities related to AMR and AMU across various sectors in Belgium, including veterinary, human healthcare, food, and the environment (Questionnaire S1). This effort resulted in a comprehensive record of their individual roles and functions.

In Questionnaire S1, participants were queried about the existence of any reports on AMR or AMU produced by the organization they represent (Questionnaire S1, Section SC for AMR and Section SD for AMU). Additionally, they were asked in which sector the report was focused (i.e., veterinary, food, human, environment) (Questionnaire S1, Questions SC2 and SD2).

In instances where their respective organizations did not publish such reports, they were prompted to share whether any other departments or services within their institute undertook this task (Questionnaire S1, Questions SC6 and SD6). Following this, participants were requested to furnish the names of these alternative services (Questionnaire S1, Questions SC7 and SD7). Furthermore, the questionnaire delved into supplementary details about these reports, encompassing aspects like the specific bacteria targeted and the frequency of their publication (Questionnaire S1, Questions SC5 and SD5). To facilitate accessibility, participants were encouraged to provide internet links to these reports if applicable (Questionnaire S1, Questions SC4 and SD4). Duplicated responses—identical answers from multiple scientists belonging to the same service—were eliminated from our analysis. The data underwent a comprehensive analysis to retain only organizations actively generating raw data or analysing results for the monitoring of AMR and AMU directly from raw data. Organizations that do not communicate results or simply compile existing reports, such as stakeholders publishing reports from time-limited scientific projects or associations providing simplified summaries for the public, were excluded from consideration.

#### 4.2.2. Step 2: Assessment of Stakeholder Positions Regarding the BELMAP Report

In step 2, the current stakeholders’ position and expectations regarding the BELMAP report were determined by three different qualitative methods [25,26,27].

- Method 1: Stakeholder expectations identification

Gathering insights into stakeholders’ future expectations during the time of data collection for the BELMAP report involved utilizing an open question (Questionnaire S1, Section SB, Question SB2) within Questionnaire S1. When analysing the results, these expectations were subsequently organized into thematic groups. This task was accomplished through the utilization of artificial intelligence (AI), specifically ChatGPT-3.5, an AI-powered language model developed by OpenAI that is capable of generating human-like text based on context. This AI is able to provide text analysis and various qualitative analyses of a transcribed discussion [28,29]. Raw data were provided to the application, accompanied by the directive to pinpoint the five most frequently occurring themes of ideas in the results, without any form of interpretation. The instruction provided to ChatGPT was “*Identify the top five prevalent themes in the provided text without interpretation or variation and give the frequencies. Please perform multiple analyses to ensure consistency in your results. If there is variability in the identified themes, redo the analysis until the results are more consistent*”. To reduce any possible variability in the answers due to the interpretation and information extraction by ChatGPT, we asked the model to perform the analysis several times and provide the best answers. Final results were also human-checked to assess quality.

- Method 2: Analysing stakeholder collaboration employing strengths, weaknesses, opportunities, and threats (SWOT) analysis

In conducting a SWOT analysis within the stakeholder analysis process (Questionnaire S1) (Questionnaire S1, Section SB, Question SB1), we aimed to evaluate the collaborative efforts of the BELMAP report’s development, ultimately contributing to its success. The rationale for employing this approach lies in its ability to identify the strengths, weaknesses, opportunities, and threats associated with the project development, enabling a comprehensive understanding of the factors that could impact its success. This analysis not only sheds light on the advantageous and challenging aspects of the collaboration, it also serves as a critical tool for addressing existing issues and making informed, strategic decisions in the context of this complex scientific endeavour.

Following the data collection, the next step concerned the recognition of analogous themes within the submitted SWOT data. This task was accomplished through the utilization of ChatGPT-3.5. Raw data was uploaded to the application, accompanied by the instruction to pinpoint the five most frequently occurring themes of ideas in the text, without any form of interpretation. The instruction provided to ChatGPT for each SWOT term was: “*Identify the top five prevalent themes in the provided text without interpretation or variation and give the frequencies. Please perform multiple analyses to ensure consistency in your results. If there is variability in the identified themes, redo the analysis until the results are more consistent*”. To reduce possible variability in results, we asked the model to perform the analysis several times and to provide the best answers. Final results were also human-checked by the first author to assess quality.

- Method 3: Focus group interview

Focus group discussions are used in qualitative research in order to facilitate group dialogue and explore the opinions of group participants on a particular topic [30]. This method was deemed useful in identifying the challenges encountered by a group of AMU/AMR scientists during the drafting of a preliminary version of the BELMAP report, and also identifying their concerns for the implementation of the full-fledged BELMAP report that would follow. The preliminary version of the BELMAP report was an internal OH AMR report, entitled the “Sciensano OH AMR report”, which was drafted by Sciensano’s collaborating scientists, as an initial activity toward the comprehensive BELMAP report. This draft (finalized in 2020) utilized exclusively internally available data. Subsequently, a focus group was conducted to interview the scientists who contributed to this report. The intention was to use this recent experience to unravel the challenges encountered during the preliminary draft and determine their expectations in the BELMAP report.

To convene this focused group, a letter elucidating the study’s aim and its voluntary nature was dispatched via email to the scientists who actively contributed to the Sciensano OH AMR internal report. Subsequently, an online unstructured focus group was organized in January 2021. Eight participants consented to take part in the activity. These were two epidemiologists, two bacteriologists representing the human and veterinary domains, a mycologist, one expert in genomic data, one person working for “Services of the managing direction” and the One Health coordinator.

The interviewer reassured the participants that the content discussed would be kept confidential at all times, and that the data would be processed without revealing their identities. Participants gave their oral informed consent, and the entire conversation was both video-recorded and transcribed *verbatim*. In order to safeguard anonymity, participants’ names were ID coded before the analysis.

Subsequent to the interview, the interviewer pinpointed the primary difficulties underscored by the participants. To ensure the accuracy of the study’s findings, the authors of the study conducted a debriefing with the interviewees via email. They discussed the *verbatim* transcriptions, addressed any uncertainties, and collected additional necessary information as needed.

#### 4.2.3. Step 3: Actor Mapping

**Figure 3:**
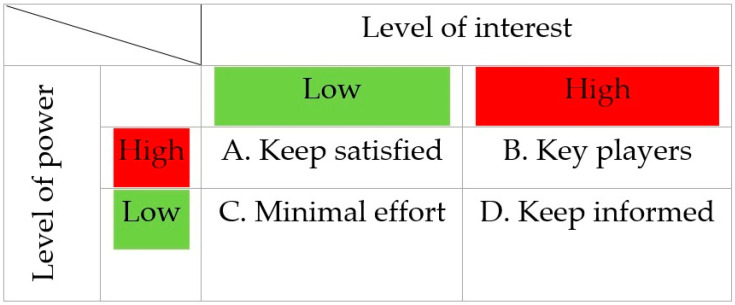
Actor-map (adapted from Mendelow).

To assess the potential impact and viability of implementing the BELMAP report, we developed an “actor map” (also referred to as the “interest-power matrix” or “Mendelow’s matrix”) [31]. Such a map presents a clear representation of the pivotal organizations and stakeholders—collectively termed as “actors”—that wield influence over the subject matter of the implementation of the BELMAP report. The actors are presented in the form of a 2 × 2 matrix combining power and interest according to how they should be managed from a project owner’s perspective (Figure 3). Power is defined as the capacity to influence decision-making. Interest is defined as the level of importance of the intervention according to the particular stakeholder, i.e., if the subject is high on the actor’s agenda.

A panel of six experts was charged with identifying the various actors with potential power and/or interest regarding the Belmap report (Table 7). These experts were affiliated with the Federal Public Health Service (n = 2), as well as Sciensano, comprising epidemiologists (n = 3) and a project manager (n = 1).

In the actor map, these distinct actors were assessed based on their power and interest dynamics, concerning the implementation of the BELMAP report. In the second questionnaire (Questionnaire S2), participants were asked to assign a score ranging from 0 to 10 for each listed actor, taking into account their respective levels of power and interest. Subsequently, the data was utilized to construct the actor map, where the mean power scores were represented on the y-axis and the mean interest scores on the x-axis. This visualization enabled the classification of each stakeholder into categories of high or low power and interest. This classification was determined by comparing their scores to the global mean power and interest scores, a method in accordance with Mendelow’s framework [31].

## 5. Conclusions

The application of stakeholder analysis in the context of implementing the national One Health BELMAP report in Belgium has emerged as a fundamental strategy. The three-step SA methodology provided insights into collaboration dynamics, stakeholder expectations, and power structures. The fragmented AMR and AMU landscape revealed in stakeholder identification highlights the need for coordination. Assessment of stakeholder positions emphasizes diverse expectations, necessitating clear communication. Actor mapping identifies key stakeholders, emphasizing strategic engagement. By identifying key stakeholders and gaining profound insights into their expectations, concerns, and motivations, this analytical approach stands as an indispensable tool for both researchers and policy-makers alike.

## Figures and Tables

**Table 1:** List of the different organizations that responded to the first questionnaire.

1	Belgian Antimicrobial Policy Coordination Committee
2	Belgian Confederation of the Dairy Industry
3	Belgian Feed Association
4	Belgian Medical Technology Association
5	Belgian Veterinary Society of Small Animals
6	Brussels Water Management Company
7	Dierengezondheidszorg Vlaanderen (Animal Health Care Flanders)
8	Federal Agency for Medicines and Health Products
9	Federal Agency for the Safety of the Food Chain
10	Federal Public Service Health
11	Federation of the Belgian Veal Sector
12	Ghent University
13	Homeopathy Belgium Industry Association
14	Institute of Tropical Medicine
15	Knowledge centre of antibiotic use and resistance in animals (AMCRA)
16	Merck Sharp & Dohme
17	National Belgian Federation of slaughterhouses, cutting plants and wholesalers for pork, bovine, sheep and Equidae
18	National Union of Christian Mutualities
19	Professional Veterinary Union
20	Public Health Administration for Walloon Region
21	Public Service of Wallonia
22	Regional Association for Animal Health and Identification a.s.b.l. (ARSIA)
23	Sciensano
24	Society of Dental Medicine
25	University of Antwerp
26	University of Liège
27	Veterinary Diagnostic Center Vedanko
28	Walloon Federation of Agriculture

**Table 2:** Summary table on antimicrobial resistance monitoring activities in Belgium.

Organization Name	Report Focus on…	Matrix/Sample	Bacterial Species	Report Is Public	Report Publication Frequency
Regional Association for Animal Health and Identification asbl	Cattle	Strains, faeces, necropsy of animals, mastitis	*Enterobacteriacae*	Yes	A report each year
			*Streptococcus* spp.		
			*Pasteurellacae*		
			*Staphylococcus* spp.		
			*Pseudomonas*		
	Chicken	Strains, faeces, necropsy of animals	*Enterobacteriacae*		
			*Streptococcus* spp.		
			*Pasteurellacae*		
			*Staphylococcus* spp.		
			*Pseudomonas* spp.		
	Pigs	Strains, faeces, necropsy of animals	*Enterobacteriacae*		
			*Streptococcus* spp.		
			*Pasteurellacae*		
			*Staphylococcus* spp.		
			*Pseudomonas* spp.		
	Small ruminants	strains, faeces, necropsy of animals, mastitis	*Enterobacteriacae*		
			*Streptococcus* spp.		
			*Pasteurellacae*		
			*Staphylococcus* spp.		
			*Pseudomonas* spp.		
	Horses	Strains, faeces, necropsy of animals	*Enterobacteriacae*		
			*Streptococcus* spp.		
			*Pasteurellacae*		
			*Staphylococcus* spp.		
			*Pseudomonas* spp.		
Dierengezondheidszorg vlaanderen	Pigs	Information not provided	*Actinobacillus pleuropneumoniae*	Yes	Information not provided
			*Bordetella bronchiseptica*		
			*Brachyspira* spp.		
			*Pasteurella multocida*		
			*Salmonella* spp.		
			*Escherichia coli*		
			*Escherichia coli* (haemolytic)		
			*Streptococcus suis*		
			*Staphylococcus aureus*		
			*Staphylococcus hyicus*		
	Chicken		*Enterococcus cecorum*	Yes	Information not provided
			*Enterococcus hirae*	Yes	
			*Enterococcus faecalis*	Yes	
			*Escherichia coli*	Yes	
			*Gallibacterium anatis*	Yes	
	Cattle		*Pasteurella multocida*	Yes	Information not provided
			*Mannheimia haemolytica*	Yes	
			*Salmonella* spp.	Yes	
			*Escherichia coli* (haemolytic)	Yes	
			*Escherichia coli*	Yes	
Sciensano	Human	Strains	*Salmonella* spp.	Yes	A report each year
			*Yersinia* spp.		
			*Shigella* spp.		
			*Mycobacteria* spp.		
			*Listeria monocytogenes*		
			*Neisseria meningitidiss*		
		Blood, Cerebrospinal fluid, Urine	*Escherichia coli*		
			*Klebsiella pneumoniae*		
			*Proteus mirabillis*		
			*Pseudomonas aeruginosa*		
			*Acinetobacter baumannii*		
			*Acinetobacter* spp.		
			*Streptococcus pneumoniae*		
			*Staphylococcus aureus*		
			*Enterococcus faecalis*		
			*Enterococcus faecium*		
		All clinical samplesScreening samples	*Staphylococcus aureus*		
			*Enterococcus faecium*		
			*Enterococcus faecalis*		
		All clinical samples	*Escherichia coli*		
			*Klebsiella pneumoniae*		
			*Pseudomonas aeruginosa*		
			*Acinetobacter baumannii*		
	Pigs	Faeces	*Enterococcus faecalis*		
			*Enterococcus faecium*		
	Veal calves	Faeces	*Enterococcus faecalis*		
			*Enterococcus faecium*		
	Laying hens	Faeces	*Enterococcus faecalis*		
			*Enterococcus faecium*		
	Breeding hens	Faeces	*Enterococcus faecalis*		
			*Enterococcus faecium*		
	Broiler chickens	Faeces	*Enterococcus faecalis*		
			*Enterococcus faecium*		
	Fattening turkeys	Faeces	*Enterococcus faecalis*		A report each year 2 years
			*Enterococcus faecium*		
	Veal calves	Pool of nasal swabs	Methicillin resistant *Staphylococcus aureus*		A report each year 3 years
	Beef cattle	Pool of nasal swabs			
	Dairy cattle	Pool of nasal swabs			
	Pigs	Pool of nasal swabs			
	Turkeys	Pool of nasal swabs			
	Broiler chickens	Pool of nasal swabs			
	Laying hens	Pool of nasal swabs			
	Pigs	Faeces	*Salmonella* spp.		A report each year
			*Escherichia coli* (extended spectrum beta-lactamase)		
			Commensal *Escherichia coli*		
			*Campylobacter* spp.		
	Cattle	Faeces	*Salmonella* spp.		
			*Escherichia coli* (extended spectrum beta-lactamase)		
			Commensal *Escherichia coli*		
			*Campylobacter* spp.		
	Chicken	Faeces	*Escherichia coli* (extended spectrum beta-lactamase)		
			*Salmonella* spp.		
			Commensal *Escherichia coli*		
			*Campylobacter* spp.		
	Food	Fresh meat broilers/pigs/beef	*Escherichia coli* (extended spectrum beta-lactamase)		A report each year
	Chicken	Faeces	*Salmonella* spp.		
	Feed	All types of feed	*Salmonella* spp.		
	Food	All types of food	*Salmonella* spp.		
Sciensano and Institute of Tropical Medicine (coordination)	Human	Strains	*Neisseria gonorrhoeae*	Yes	A report each year

**Table 3:** Summary table on antimicrobial usage monitoring in Belgium.

Organization Name	Report Focus on…	Report Is Public	Report Publication Frequency
Knowledge centre of antibiotic use and resistance in animals (AMCRA)	Livestock pigs, veal calves, poultry	Yes	A report each year
Sciensano	Long-term care facilities (mainly nursing homes)	Yes	A report each 3 years (point prevalence survey)
	Acute care hospitals	Yes	A report each 5 years (point prevalence survey)
Sciensano	Hospitals	Yes	The reports can be downloaded by the hospitals at any time after validation of their data. Individual hospital reports exist for the years 2015, 2017 and 2019 with Belgian benchmark data
UGent	All animals (livestock and pets)	Yes	A report each year

**Table 4:** Main stakeholder expectations regarding the BELMAP report.

Theme	Description
Collaboration and partnerships	This theme emphasizes the need for cooperation and alliances between different sectors, stakeholders, and experts in both human and veterinary medicine. The goal is to collectively address antibiotic use and resistance, ensuring a comprehensive approach that involves governments, institutions, professionals, and the general public.
Data collection and analysis	The focus here is on gathering comprehensive data related to AMU and AMR. This involves compiling information from various sources, including human and animal health sectors, different geographical levels (national, regional, local), and potentially environmental factors. Analysing this data is crucial for informed decision-making.
Clear guidelines and recommendations	This theme highlights the importance of developing explicit and actionable guidelines and recommendations. These guidelines can provide directions for responsible antibiotic use, both in human and veterinary medicine. The aim is to offer practical, evidence-based guidance that helps in reducing unnecessary antibiotic use and slowing down the development of antibiotic resistance.
Awareness and education	This theme underscores the significance of creating awareness and educating both professionals and the general public about antibiotic resistance. The goal is to enhance understanding of the issue’s severity and the importance of responsible antibiotic use. Through education, individuals can make informed decisions and support the overall efforts to combat antibiotic resistance.
Practical implementation	This theme revolves around putting strategies into action. It involves translating guidelines, recommendations, and awareness efforts into practical steps. Implementing changes in antibiotic use practices, transitioning from group treatments to individualized treatments, exploring alternatives to antibiotics, and incorporating diagnostic tools are among the practical measures discussed.

**Table 5:** This table illustrates the top five prevalent themes identified by artificial intelligence in relation to the strengths (S), weaknesses (W), opportunities (O), and threats (T) associated with the BELMAP report.

	Strengths	Weaknesses	Opportunities	Threats
1	Collaboration (9)	Fragmentation of competencies (6)	Increasing awareness of antimicrobial resistance (AMR) (8)	Resource limitations and underfunding (10)
2	Data sharing and harmonization (5)	Slow decision processes (6)	One health approach and collaboration (7)	Challenges in data collection and harmonization (9)
3	One health approach (5)	Challenges in data harmonization and sharing (3)	Harmonization and alignment (6)	Impact of COVID-19 on priorities and resources (2)
4	Awareness and communication (4)	Commercial and economic interests (2)	Integration of human, animal, and environmental domains (3)	Complexity and potential overload (2)
5	Expertise and expert involvement (4)	Differences in stakeholder agreements (2)	Innovative therapies and research collaborations (3)	Competition and comparison to other tnitiatives (2)

Legend. The numbers in parentheses indicate the absolute frequency of mentions.

**Table 6:** Main expectations and concerns regarding the BELMAP report identified during the focus group.

Theme	Description
Involvement and participation	The dialogue commenced with a consideration of effective stakeholder involvement. The consensus was that clear mandates and missions are essential to acquire authority in engaging stakeholders.
Mandate and authority	Participants highlighted the significance of obtaining an official mandate to underscore the credibility and seriousness of the reports’ objectives. They emphasized that an official mandate, as reflected in the National Action Plan, would also ensure the allocation of necessary resources and funding. The conversation further delved into the need for clarity on the distribution of resources, leadership roles, and funding allocation.
Collaboration and data comparison	Collaboration among stakeholders emerged as a focal point. While Sciensano held some data, participants stressed the importance of involving various stakeholders to incorporate diverse perspectives. Challenges were identified in linking data across different sectors for meaningful comparison. The complexities of aligning methodologies, particularly in comparing data collection practices between animals and humans, were highlighted. The discussion acknowledged the challenges of comparability, especially due to variations in monitoring practices.
Defining content and scope	The dialogue also touched upon the significance of predefining the report’s content given the iterative nature of the project. Stakeholders emphasized the importance of outlining what is feasible for reporting within a One Health framework. It was suggested that discussions should encompass data collection methodologies and protocols to facilitate meaningful comparison.
Short and policy-guiding report	Participants clarified that the primary objective is to craft a concise, policy-guiding report that succinctly summarizes relevant data and trends. The focus is on assembling pertinent data rather than establishing an extensive surveillance system.

**Table 7:** List of the different stakeholders having potential power and interest regarding the Belmap report.

1	Belgian Antibiotic Policy Coordination Committee (BAPCOC)
2	Belgian Feed Association
3	Certification/label/sector/professional associations
4	Consumers
5	Farmers association
6	Federal agency for medicines and health products
7	Federal Public Service
8	Food Agency for the Safety of the Food Chain
9	Food retailers
10	Knowledge centre of antibiotic use and resistance in animals (AMCRA)
11	Medical doctors
12	Milk sector organisations/labs
13	Minister of Agriculture
14	Minister of Health
15	National Belgian Federation of slaughterhouses (FEBEV)
16	National Institute for Health and Disability Insurance (INAMI/RISIV)
17	Pharma industry
18	Risk assessment group
19	National reference laboratory (Sciensano)
20	Universities
21	Vet association
22	Vet practitioners
23	Vet regional laboratory 1 (Association régionale de santé et d’identification animales)
24	Vet regional laboratory 2 (Dierengezondheidszorg Vlaanderen)

## Data Availability

The entire raw data are available on request from the corresponding author, after anonymisation and if participants anonymity and confidentiality can be guaranteed.

## Supplementary Materials

The following supporting information can be downloaded at: https://www.mdpi.com/article/10.3390/antibiotics13010084/s1, Questionnaire S1; Questionnaire S2; Results S1: Stakeholders’ expectations regarding the nation One Health report; Results S2: Focus group
