# Supplementary material for: Using a Stakeholder Analysis to Implement the Belgian One Health National Report for Antimicrobial Use and Resistance
Source: Antibiotics (Basel). 2024 Jan 16;13(1):84. doi: 10.3390/antibiotics13010084 (PMC10812551; doi:10.3390/antibiotics13010084)
Supplement: Supplementary file 1 [file antibiotics-13-00084-s001.zip › antibiotics-2775441-supplementary.pdf]

Supplementary Materials

Questionnaire S1

The One Health European Joint Programme (OHEJP) aims to align European countries through a joint priority setting in the domains of foodborne zoonosis, antimicrobial resistance and emerging threats, and joint programming of research agendas. This is a considerable opportunity for harmonization of approaches, methodologies, databases and procedures for the assessment and management of foodborne hazards, emerging threats and antimicrobial resistance across Europe, which will improve the quality and compatibility of information for decision making.

The One health suRveillance Initiative on harmOnization of data collection and interpretation (ORION) is a OHEJP project that aims at establishing and strengthening inter-institutional collaboration and transdisciplinary knowledge transfer in the area of surveillance data integration and interpretation, along the One Health objective of improving health and well-being. This will be achieved through an interdisciplinary collaboration of 13 veterinary and/or public health institutes from 7 European countries.

This survey focuses on the Pilot project for Belgium, part of the "One Health Surveillance Knowledge Hub" (ORION work package 2). In short, this Pilot project takes place in a context the federal and regional Belgian authorities require to develop a national One Health antimicrobial resistance (AMR) action plan. In this goal, the first step is to develop a yearly national One Health AMR report. The development of this report will require collaboration and coordination of the actions taken by the many involved partners (Belgian Health Care Knowledge Centre, 2019). The ORION pilot project will help to overcome the possible challenges that could happen during the process.

<u>Study expectations</u>

You are expected to fill this online questionnaire and another one some weeks later. The time required to fill the first questionnaire is 10-20 minutes and 10 minutes for the second. If you face any difficulty or have a question, you are free to contact the investigator.

<u>Rights for refusal, access to personal information</u>

Your decision to participate in this study is completely voluntary. You may withdraw from your participation at any time for free.

<u>Benefits Associated with Study Enrollment</u>

People will be informed on the final results of the pilot project.

Use of results; individual return or not

By signing this form, you authorize the use and disclosure of your data and findings during the course of this study for publication and presentation.

Confidentiality / privacy

We guarantee that absolute confidentiality and anonymity. Your personal data will be only use to contact you if needed. No IP addresses will be collected.

Only the scientists in the Veterinary Epidemiology unit (Sciensano) have access to the data. Your personal data will never be transmitted to anyone else.

Each participant will be identified on the questionnaires by an individual ID in order it will be impossible for anyone to identify them. Only the primary investigator will know what your ID is. Your personal data (name, first name, e-mail) will never be directly associated with the questionnaires. We will not be sharing any information to anyone outside of the research team.

Long-term storage

During and after the study, all information collected will be securely stored on a secure internet server. Data will be stored during a period of 10 years but you have the right to have all information collected about you modified or deleted at any moment for free. After this period of time, the data will be destroyed

To continue please first accept our survey data policy. ☐

Section SA: Personal data

SA1. What is your organization's name?

SA2. Where is your organization located?

SA3. What is your function?

Section SB: SWOT analysis and expectations

SB1. Belgium is about to write a national One Health antimicrobial report. Considering all institutions actively involved in this topic should collaborate, cite at least one strength, one weakness, one opportunity and one threat related to this project

Please write your answers here:

S*trengths*: characteristics of the project that give it an advantage over others.

W*eaknesses*: characteristics of the project that place it at a disadvantage relative to others.

Opportunities: elements in the environment that the project could exploit to its advantage.
Threats: elements in the environment that could cause trouble for the project.

SB2. What are your expectations on the future One Health national antimicrobial report?
Please write your answer here:

Section SC: Antimicrobial report

SC1. Does your service publish a report on antimicrobial resistance? Yes/No
SC2. This report focuses on antimicrobial resistance in feed/ Food (from animal origin)/ Food (not from animal origin)/ Live animals (livestock)/ Other live animals (i.e. pets)/ Environment/Humans/ Other (give precisions….)
SC3. Is the report public? Yes/No
SC4. Can you provide the internet link where the report can be found?
SC5. Can you give more details on the frequency the report is published (e.i 1 x/year), on the bacteria and on the antimicrobials tested? If this information is available on a public report and you have already provided the link, just write: see report.
SC6. Does at least one other service in your institute publish a report on antimicrobial resistance? Yes/No
SC7. Can you cite the name(s) of these service(s)?

Section SD: Antimicrobial usage or consumption report

SD1. Does your service publish a report on antimicrobial usage or consumption? Yes/No
SD2. This report focuses on antimicrobial usage or consumption in feed/ Food (from animal origin)/ Food (not from animal origin)/ Live animals (livestock)/ Other live animals (i.e. pets)/ Environment/Humans/ Other (give precisions….)
SD3. Is the report public? Yes/No
SD4. Can you provide the internet link where the report can be found?
SD5. Can you give more details on the frequency the report is published (e.i 1x/year), on the bacteria and on the antimicrobials tested? If this information is available on a public report and you have already provided the link, just write: see report.
SD6. Does at least one other service in your institute publish a report antimicrobial on usage or consumption? Yes/No
SD7. Can you cite the name(s) of these service(s)?

Questionnaire S2.

Study expectations

You are expected to fill this online questionnaire The time required to fill the first questionnaire is 10-20 minutes. If you face any difficulty or have a question, you are free to contact the investigator.

Rights for refusal, access to personal information

Your decision to participate in this study is completely voluntary. You may withdraw from your participation at any time for free.

Benefits Associated with Study Enrollment

People will be informed on the final results of the pilot project by sending their e-mail address at mickael.cargnel@sciensano.be.

Use of results; individual return or not

By signing this form, you authorize the use and disclosure of your data and findings during the course of this study for publication and presentation.

Confidentiality / privacy

We guarantee that absolute confidentiality and anonymity. No IP addresses will be collected.

Only the scientists in the Veterinary Epidemiology unit (Sciensano) have access to the data.

Each participant will be identified on the questionnaires by an individual ID in order

To continue please first accept our survey data policy. ☐

Actor map.

SB1. Can you grade these stakeholders based on their power and interest in publishing a common national one health antimicrobial/antimicrobial usage or consumption report?

Power= the capacity to influence the decision-making, the ability to decide upon implementing and how to implement the intervention. 0= no power and 10= the maximum power

Interest=the level of importance the intervention has to the particular stakeholder, if the subject is high on the stakeholder's agenda. 0= no interest

and 10= the maximum interest

Power

	0	1	2	3	4	5	6	7	8	9	10
Food Agency for the Safety of the Food Chain (AFSCA/FAVV)	☐	☐	☐	☐	☐	☐	☐	☐	☐	☐	☐
Sciensano	☐	☐	☐	☐	☐	☐	☐	☐	☐	☐	☐
Association régionale de santé et d'identification animales (ARSIA)	☐	☐	☐	☐	☐	☐	☐	☐	☐	☐	☐
Dierengezondheidszorg Vlaanderen (DGZ)	☐	☐	☐	☐	☐	☐	☐	☐	☐	☐	☐
INAMI/RISIV	☐	☐	☐	☐	☐	☐	☐	☐	☐	☐	☐
Pharma industry	☐	☐	☐	☐	☐	☐	☐	☐	☐	☐	☐
BAPCOC	☐	☐	☐	☐	☐	☐	☐	☐	☐	☐	☐
Universities	☐	☐	☐	☐	☐	☐	☐	☐	☐	☐	☐
AMCRA	☐	☐	☐	☐	☐	☐	☐	☐	☐	☐	☐
Risk assessment group (RAG)	☐	☐	☐	☐	☐	☐	☐	☐	☐	☐	☐
Medical doctors	☐	☐	☐	☐	☐	☐	☐	☐	☐	☐	☐
Vet practitioners	☐	☐	☐	☐	☐	☐	☐	☐	☐	☐	☐
Farmers association (FWA, boerenbond,...)	☐	☐	☐	☐	☐	☐	☐	☐	☐	☐	☐
Vet association (Union professionelle vétérinaire VDV,...)	☐	☐	☐	☐	☐	☐	☐	☐	☐	☐	☐
Federal Public Service (SPF/FOD)	☐	☐	☐	☐	☐	☐	☐	☐	☐	☐	☐
Minister of Health	☐	☐	☐	☐	☐	☐	☐	☐	☐	☐	☐
Minister of Agriculture	☐	☐	☐	☐	☐	☐	☐	☐	☐	☐	☐
Consumers	☐	☐	☐	☐	☐	☐	☐	☐	☐	☐	☐
FEBEV (Fédération Belge de la Viande/De Federatie van het Belgische Vlees)	☐	☐	☐	☐	☐	☐	☐	☐	☐	☐	☐

Interest

	0	1	2	3	4	5	6	7	8	9	10
Food Agency for the Safety of the Food Chain (AFSCA/FAVV)	☐	☐	☐	☐	☐	☐	☐	☐	☐	☐	☐
Sciensano	☐	☐	☐	☐	☐	☐	☐	☐	☐	☐	☐
Association régionale de santé et d'identification animales (ARSIA)	☐	☐	☐	☐	☐	☐	☐	☐	☐	☐	☐
Dierengezondheidszorg Vlaanderen (DGZ)	☐	☐	☐	☐	☐	☐	☐	☐	☐	☐	☐
INAMI/RISIV	☐	☐	☐	☐	☐	☐	☐	☐	☐	☐	☐
Pharma industry	☐	☐	☐	☐	☐	☐	☐	☐	☐	☐	☐
BAPCOC	☐	☐	☐	☐	☐	☐	☐	☐	☐	☐	☐
Universities	☐	☐	☐	☐	☐	☐	☐	☐	☐	☐	☐
AMCRA	☐	☐	☐	☐	☐	☐	☐	☐	☐	☐	☐
Risk assessment group (RAG)	☐	☐	☐	☐	☐	☐	☐	☐	☐	☐	☐
Medical doctors	☐	☐	☐	☐	☐	☐	☐	☐	☐	☐	☐
Vet practitioners	☐	☐	☐	☐	☐	☐	☐	☐	☐	☐	☐
Farmers association (FWA, boerenbond,...)	☐	☐	☐	☐	☐	☐	☐	☐	☐	☐	☐
Vet association (Union professionelle vétérinaire VDV,...)	☐	☐	☐	☐	☐	☐	☐	☐	☐	☐	☐
Federal Public Service (SPF/FOD)	☐	☐	☐	☐	☐	☐	☐	☐	☐	☐	☐
Minister of Health	☐	☐	☐	☐	☐	☐	☐	☐	☐	☐	☐
Minister of Agriculture	☐	☐	☐	☐	☐	☐	☐	☐	☐	☐	☐
Consumers	☐	☐	☐	☐	☐	☐	☐	☐	☐	☐	☐
FEBEV (Fédération Belge de la Viande/De Federatie van het Belgische Vlees)	☐	☐	☐	☐	☐	☐	☐	☐	☐	☐	☐

Results S1

Stakeholders' expectations regarding the nation One Health report

ID	Answers
1	Initially to provide a yearly or two-yearly (depending on the feasibility) report on :<br>- antimicrobial use in three main sectors (human, animal and environment)<br>- antimicrobial resistance in three main sectors (human, animal and environment)<br>On a later stage to provide also analyses on the links existing among the three sectors.<br>The report should have a large visibility in order to provide a clear communication and information to the scientific community on the subject.
2	reach useful and workable conclusions on the field to improve AMR and to convince the field to participate actively by using more diagnostic and prevention => practical and not theory
3	I have no particular expectations. I hope it will contain good recommendations but only if there are also sufficient financial means to implement the recommendations will it also lead to change.
4	Reporting of baseline figures on antibiotic use and resistance in the outpatient and inpatient setting, pet and vet sector, environment and it's relation or association with AMR. This would require statistical analysis.
5	Clear strategy and goal setting<br>realistic plan<br>clear description of responsibilities<br>clear demand for finance towards government
6	Prevention of contamination, prevention of worsening<br>On time reducing unnecessary antibiotic use by the use of point of care tests, reducing antibiotic resistance on the long term, starting with antibiotics when really needed
7	joint data about human and veterinary sector<br>recommendations for different stakeholders
8	Scientific based report, but with simple and correct 'take home messages'
9	"Awareness raising in both veterinary and human medicine" only antibiotics and it must be"<br>Reduce resistance through responsible antibiotic use<br>Strong incentive to adjust management in livestock farming and find alternatives to antibiotics<br>Need to finish group treatments and over go to individual treatments"
10	It is important to have clear agreements at the start of the project. Maybe focus on selection of bacteria and the molecules studied. The report should give a benefit to the people who share data (PT organization) but most import it should give a clear message to the people in the field. Raising awareness of the right target groups in the field by explaining the results in various forums.<br>The collection and analysis of the data should be performed by one institute but data should be discussed before publication by different experts of the different disciplines involved (use of AB, lab testing, statistical, ...) It should be not a report only for scientist but also for people involved in the global goal "lower use of AB and lowering the resistance in general
11	The report should compile the AMU and AMR data for as well the animal health sector as the human health sector. This as well on national level, regional level as on more local level (cities or areas).
12	Monitoring and results

13	the collaboration of different institutions to achieve the defined goals
14	Guidance document on how to reduce the use of antibiotics, supported and endorsed by the national authorities
15	Data available to support policy<br>Scientific conclusions can be taken
16	one health approach, we should not be confined to the use of antibiotics in breeding. the unreasonable use of antibiotics is known in pets who are in close contact with their owner.
17	To obtain insights in how antibiotic use in humans or in animals influence antibiotic resistance in animals and humans, respectively<br>To see the evolution of the resistance for different strains<br>To get an update on alternatives
18	that it is concise, interesting, no more than 30 pages, with a good summary and most important it should be ready within 6 months after the end of the year
19	I have no real expectations of the report. I hope it contains some concrete recommendations but if there are insufficient means to implement these recommendations it will change nothing.
20	Propose an all-integrated plan to attack AMR.<br>In that plan, we would like to consider the possibility and encourage the research for alternative solutions to lower the overuse of antibiotics
21	Tools to change what is necessary. Full dedication of the government. Time/money dedicated. Follow up on rules. Timeframe of plans.
22	one reference plan for the whole country with enforceable measures
23	It should increase awareness around AMR for every stakeholder in order to also allocate more budget for this problem<br>It should enhance private and public partnership and dialogue
24	it will have an impact on the use and prescription of antibiotics but will need perseverance in implementation
25	That all data that are collected in different Institutions can be merged and different domains of expertise can work together in order to come to one strategic 'One Heath' project, tackling the rise of emerging pathogens.
26	My expectations on the report: Should be a priority for the group working on it. (sufficient time should be awarded) Report ready on time so that measures can be taken rapidly. Ideally not a static report but dynamic eg on a website with regular updates. Should be an integrated report, not just figures per sector but links between sectors should be integrated.
27	The hope that more will be invested in NGS-based surveillance
28	A global picture of the AMR situation in all the environmental settings and possible interactions.
29	Get a better insight in the putative impact of AMU in one sector on AMR in this sector or on AMR in other sectors. If associated with a funding of whole genome analyses, the comparison of human and animal strains could highlight AMR genes or AMR bacterial clones circulating in and between the different sectors.
30	Guidelines in order to assist health specialists to manage antibioresistance and to adapt to new methods

31	I hope it will show the participation of all partners involved in the AMR action plan. Above all, the cross-sector interpretation of the data should be possible (encouraged). Therefore, the support of the authorities is needed (to overcome barriers)
32	Providing recurrent harmonized presentations of the results allowing for<br>- follow-up over time,<br>- detection of increased use of critical antibiotics<br>- identify causative link consumption/resistance<br>- readable also for non-experts => facilitate communication
33	at a first step, an overview of the currently available data of resistance in different species and a description of what is missing or what could be added in the future. I hope this work will help in harmonizing further the approaches and methods used in surveillance of AMR in different sectors, as much as this is realistically feasible. In this way, links between different sectors could be made based on a sufficient body of knowledge
34	to have an overview of the situation in Belgium concerning AMR and its evolution, to know if the levels defined for the different indicators are reached or not, to have a global picture, from different fields/domains/approaches of the issue
35	to have a centralized report that allow everyone to get easier access to the results
36	Clear Guidelines destined to all professionals and general public alike
37	wide circulation of the report to make policies aware of the existing threat
38	competence and realism, therefore confrontation and listening to actors in the field
39	It should question the model of industrial farming and the conditions of emergence of AMR issues (lack of genetic diversity, intensification of production and overpressure on farm animals), including when such policies are going against strongly instituted interests such as the one of farming unions.
40	Description of successes in animal use of antibiotics
41	That it will start from a shared responsibility of both human and veterinary medicine. Too often it seems that only animal production is designated as the sector in which further efforts must be made, whereby I believe that also in human medicine, there is much progress that can be made. Furthermore the different government levels have to be involved and achievable targets have to be preceded.

This table displays for each respondent (ID), the raw stakeholders' expectations regarding the future national One Health antimicrobial report (BELMAP) (column 'answer').

Results S2

Focus group

Introduction to the project

Mail sent to the participants before the meeting

Dear colleagues,

Sciensano is involved in a One Heath European Joint Programme (ORION, pilot project for BE). One goal is to help Belgium to better implement its

national OH AMR report. Last year, we wrote a OH Sciensano report. It would be interesting for the project but also for the future report, to highlight the difficulties that we have faced when we wrote it and to anticipate the (potential) upcoming one.
To do so, we propose a virtual focus group (10 minutes) to talk about. Please note that the discussion will be recorded (more information on confidentiality below). We consider that if you participate in the focus group, you have read the information and agree.

The One Health European Joint Programme (OHEJP) aims to align European countries through a joint priority setting in the domains of foodborne zoonoses, antimicrobial resistance and emerging threats, and joint programming of research agendas. This is a considerable opportunity for harmonisation of approaches, methodologies, databases and procedures for the assessment and management of foodborne hazards, emerging threats and antimicrobial resistance across Europe, which will improve the quality and compatibility of information for decision making. The One health suRveillance Initiative on harmOnization of data collection and interpretation (ORION) is a OHEJP project that aims at establishing and strengthening inter-institutional collaboration and transdisciplinary knowledge transfer in the area of surveillance data integration and interpretation, along the One Health objective of improving health and well-being. This will be achieved through an interdisciplinary collaboration of 13 veterinary and/or public health institutes from 7 European countries. This survey focuses on the Pilot project for Belgium, part of the "One Health Surveillance Knowledge Hub" (ORION work package 2). In short, this Pilot project takes place in a context the federal and regional Belgian authorities require to develop a national One Health antimicrobial resistance (AMR) action plan. In this goal, the first step is to develop a yearly national One Health AMR report. The development of this report will require collaboration and coordination of the actions taken by the many involved partners (Belgian Health Care Knowledge Centre, 2019). The ORION pilot project will help to overcome the possible challenges that could happen during the process.

Rights for refusal, access to personal information

Your decision to participate in this study is completely voluntary. You may withdraw from your participation at any time for free.

Benefits Associated with Study Enrollment

People will be informed on the final results of the pilot project.

Use of results; individual return or not

You authorize the use and disclosure of your data and findings during the course of this study for publication and presentation.

Confidentiality / privacy

We guarantee confidentiality and anonymity. Your name will not be cited, but we can cite the name of your service in the introduction to define the different profiles that took part in the focus group. The discussion will be recorded and will be transcribed verbatim.

Only the scientists in the Veterinary Epidemiology unit (Sciensano) have access to the raw data. Raw record (with your name) will never be transmitted to anyone else.

Each participant will be identified by an individual ID in order it will be impossible for anyone to identify them. Only the primary investigator will know what your ID is. We will not be sharing any information to anyone outside of the research team.

Long-term storage

During and after the study, all information collected will be securely stored on a secure internet server. Data will be stored during a period of 10 years but you have the right to have all information collected about you modified or deleted at any moment for free. After this period of time, the data will be destroyed.

Declaration of Competing Interest

The positions and opinions presented reflect the interviewees' opinions and are not intended to represent the views or scientific works and opinions of Sciensano.

Participants

Veterinary epidemiology 1

Veterinary epidemiology 2

Services of the managing direction

Food pathogens

Bacterial diseases

Veterinary bacteriology

Mycology and aerobiology

One health coordinator

I: Interviewer

Glossary

NRC: national reference center

Date: 14/01/2021

Transcript

I: This is an open discussion. There is no specific question. I would like to give you a free space to discuss, first, the Sciensano report and especially the problems we have faced.

ID1: Ok.

I: I don't know if you can begin, ID1?

ID1: I have started recording.

I: So who would like to begin? I don't know, for example, for example, I know it was very difficult to involve …to involve people. So perhaps, do you think it would have been more relevant to create a Doodle for a better participation, for example?

ID1: You mean for this working group for this…

I: For the last Sciensano report. As you know it was quite difficult. It was written in a hurry in the last week because everyone was involved in different tasks. So do you think it would be a better solution to organize the work between us?

ID1: Uh. Yeah. Uh, depending on how many people you are, I think it is a good idea, no?

ID2: Yes, but I think that to do so, we need some visibility in the population of people who has to answer the questions. So we need a mandate first and a clear mission. And with that authority, we can contact the people and a long time before they have to submit something, just to tell them what we expect and what is the… submit them a list of questions. So indeed, it's necessary to organize the conversations, define the contents and ask specific questions because the… the whole project will work on iterations. So a first general report and then more precise information collected and more precise back information shared with the stakeholders. So indeed, we need a first deeper thinking about what we want to collect as information and what we want to have in the report and so we can organize the whole project on a very long time.

ID1: But I don't think that this should be only decided by us. It should be in conversation with all people…with all the people involved. So, um, because we have some data also at Sciensano, but we don't have many other data that we'd like to include. So I think we should be, uh, should reach out also to as many people as possible. This was not possible, of course, the last year because of the COVID and the lack of time but I think this will be possible this year.

I: So do you think the authority should be more present to address, to say what to do?

ID2: Yes, I suppose so. We are wanting to do this report that, uh, um, yeah for uh, for Belgium but if we don't have an official mission, why would people think we are serious and wanting to do something useful? So I think, yes, we need a mandate from an authority. What we're seeing on the goodwill of person and what is the representatively of goodwill?

ID1: This mandate is defined in the National Action Plan, which will be approved, hopefully… So this will be approved. So the mandate will be given and there will be financing coupled with this also.

ID2: It's still not clear who will receive this money and which team will lead the project. So we asked for money and people, but there is no official document at that stage that we will receive these resources.

ID1: No. No, because the action plan has not yet been approved. Hmm.

I: So, first the stakeholders should be clearly defined and then to clearly define who will receive the money. That's what you are saying? At the moment, the stakeholders are not clearly defined?

ID2: We have our imagination and we imagine the content that should be answering the questions these stakeholders are asking themselves or asking us, but indeed there must be a debate on the content and on the aim.

I: ID2 and ID3, do you want to add something in this discussion?

ID3: Yes, I agree with the others. The first thing is to officially have a leader and we have to propose to all the stakeholders, the aims that we think about and the.. (*audio was interrupted*) …the first listed aims. I don't think we should begin with all the stakeholders without having a plan to present. I think we should draft a first plan based on the previous short report that we did last year and think what we have to add and then submit it to the other stakeholders and discuss it together.

ID2: And ask them what they want to see in the report and how we can improve it.

ID4: I think I agree with you, both, and I think we have to predefine the content of this report based on what we have… on what we have done before and the

problems that we had. It was very difficult to find the target bacteria that we wanted to include and to have to data from the different sectors. So we have first to predefine what is possible to report in a One Health perspective because I think there were a lot of gaps this year and it was difficult to compare the data. So we need maybe first to predefine the content and to see what is the most feasible to do.

I: But who has to do that? Sciensano or the authority?

ID4: I was thinking Sciensano together with the stakeholders to see what are the data that we have and what we can do with these data. Because (*noise*) national control plan, there are very well regulated and we have very constant data yearly. But the data that we have from to compare with the human part is very difficult. And it was like to do something this year to propose a one health report, but it was… we had a very little points of comparison to do and there was a lot of data that were not included because it was impossible to compare.

ID3: Yes, indeed. There are lots of differences between the monitoring in different sectors and for the moment with the current monitoring it is difficult to compare and so it's difficult to have a real one health report.

ID1: But if you… it's… it's not so difficult to define what you don't want to include from the veterinarian and food side, right? Because you monitor indicators. That's all data you have. I would not consider it very difficult. I would only consider that the most difficult part is indeed how to link the *staph aureus* and the *campylobacter* with what you see at the human side. But that is something which needs to be discussed not with us, because we don't have the data. That needs to be discussed with the CNR of *campylobacter* and the CNR of *staph aureus*. So that cannot be decided by us and I think this should be discussed

ID4: Or maybe the lead has to be taken by the NRC, not from us, because we have a very small amount of data compared to them.

ID1: We should not forget that the purpose of this report is to have 10 to 15-page document, a very, very short which summarizes all the other reports that we are doing, which can have a large reference section. But the goal is to have a policy guiding small, small document. Right. So we should not be looking at the hundred page documents. It will be a very short report which just assembles the relevant data. So. Yeah, yeah, that is the goal. So all the other reporting will still be going on. This will just be some kind of summary. So I do believe that the stakeholder meetings with the externals are of crucial

importance, because in these meetings, we just have to decide what is relevant and what's not to have policy guiding.

ID2: Ok, but for me, one added value of this report was to follow up in parallel the consumption and resistance both in humans and animals.

ID1: Yes, this will be included, hopefully.

ID2: Yes, but try to have the information to identify causative link. And that would be done over time, and that makes necessary to harmonize the data collection and defining a way of analyzing data. So it goes further than just having the previous report all gathered in the same document.

ID1: No, I think this will be the purpose of the EVARESIT project, no? This is what the personal work on?

ID2: The EVARIST project focuses on the way of describing the events of antimicrobio resistance in Belgium and to provide guidelines. I would say it's just guidelines and not the definition of the content of the common report. So if you want to define a causative link between consumption and resistance, we need extra data collected. It's not the same as surveillance.

ID1: So, but it's really done at the European level, no? There are already two reports doing this. What about these reports again? Where they collect trends and resistance in rapport to consumption. So we just have to adjust, of course, we need to apply the same statistical methods, I think, that are used in these reports. It's not the purpose of this report to organize a new surveillance system, to organize new data collection. That is really not the purpose. There is also no new funding or no personnel or whatever to do this.

ID3: Yes, but then we will be limited in the scope that we can discuss because, for example, for *staphylococc*i, the context of the bacterial collection is very different between the NRC humans and our team. So it was really difficult to compare because the context is not the same. And you cannot compare.

ID1: No, but we just have to acknowledge them. Yeah. Yeah.

ID3: So it's the first step to list what is comparable, what is not. And we did this exercise last year, but we will not be able to go deeper because if the monitoring doesn't change, we will not be able to do a more complete report.

ID1: No, no. Yeah, that's true. It's not the purpose of this support to change the monitoring. It is just to compare what is possible and to acknowledge the limits if needed. But this needs to be in discussion with all the stakeholders, of course.

ID4: Maybe it will be useful to us, to the stakeholders, how they do the monitoring. I mean, how they do the monitoring, the collection of the isolates, what is the protocol for them and to see if there is a possibility to compare or we just don't take care about these collection methods and we take care about the outcome of the resistance profiles. Because it is impossible to compare in our case the surveillance and in the case of humans, I don't know. Is there also a surveillance or only clinical cases?

ID1: Well, I don't know. Maybe there are, for example, maybe there are surveillance studies for staph aureus with healthy volunteers. I don't know. That would be comparable I think. We have to be able to ask the people from the NRC.

ID3: Last year it was not the case.

ID1: I don't know their plans (*laughs*).

ID3: I don't know for the future but last year it was not the case.

ID1: We have to ask.

ID4: I think there is a surveillance one year every two years or something like this but… I don't know…

I: Ok, so if I summarize the ideas. The first thing is to ask the authority on what they expect and then to have a good discussion with all the stakeholders to know what is available and then to come back to the authority to see if they agree on to the plan we are writing based on the what the pathogens and the bacteria availabilities. Is it is it right?

ID2: Yes, yes. Yes for me…

I. Time is running out. I don't want to be too long. So do you want to add something else?

ID5: I want to say something. I was disconnected for 10 minutes because there was no I have really bad connection today. So in case you talk to me, I didn't reply. It was because I was trying to reconnect. So I'm sorry for that.

I: And have you heard the conversation?

ID5: I think I've heard half of the discussion.

I: Do you want to add something else? Something to close the debate?

ID1: I agree with the conclusion: first to contact the authority and then after I see the feasibility for the stakeholders.

I: Ok. I would like to thank you for your participation and the time you have given to me.
